# Prevalence of breast muscle myopathies (spaghetti meat, woody breast, white striping) and associated risk factors in broiler chickens from Ontario Canada

**DOI:** 10.1371/journal.pone.0267019

**Published:** 2022-04-15

**Authors:** Sunoh Che, Chaoyue Wang, Csaba Varga, Shai Barbut, Leonardo Susta

**Affiliations:** 1 Department of Pathobiology, University of Guelph, Guelph, Ontario, Canada; 2 Department of Food Science, University of Guelph, Guelph, Ontario, Canada; 3 Department of Pathobiology, University of Illinois at Urbana Champaign, Champaign, IL, United States of America; Foshan University, CHINA

## Abstract

Spaghetti meat (SM), woody breast (WB), and white striping (WS) are myopathies that affect the pectoral muscle of fast-growing broiler chickens. The prevalence and possible risk factors of these myopathies have been reported in other countries, but not yet in Canada. Thus, the objective of this study was to assess the prevalence and risk factors associated with these myopathies in a representative population of Canadian broilers. From May 2019 to March 2020, 250 random breast fillets from each of 37 flocks (total, 9,250) were obtained from two processing plants and assessed for the presence and severity of myopathies. Demographic data (e.g., sex and average live weight), environmental conditions during the grow-out period (e.g., temperature), and husbandry parameters (e.g., vaccination) were collected for each flock. Associations between these factors and the myopathies were tested using logistic regression analyses. The prevalence of SM, severe WB, and mild or moderate WS was 36.3% (95% CI: 35.3–37.3), 11.8% (95% CI: 11.2–12.5), and 96.0% (95% CI: 95.6–96.4), respectively. Most (85.1%) of the fillets showed multiple myopathies. Regression analyses showed that the odds of SM increased with live weight (OR = 1.30, 95% CI 1.01–1.69) and higher environmental temperature during the grow-out period (OR = 1.75, 95% CI 1.31–2.34). The odds of WB increased with live weight (OR = 1.23, 95% CI 1.03–1.47) and when flocks were not vaccinated against coccidia (OR = 1.86, 95% CI 1.51–2.29). This study documents for the first time a high prevalence of myopathies in Ontario broilers, and suggests that these lesions may have a significant economic impact on the Canadian poultry industry. Our results indicate that environmental conditions and husbandry are associated with the development of breast myopathies, in agreement with the current literature. Future studies are needed to determine how risk factors can promote the occurrence of these conditions, in order to implement possible mitigating strategies.

## Introduction

During the past decade, a group of myopathies has emerged in intensively reared broiler chickens, which primarily affect the *Pectoralis major* muscle (pectoral muscle). Such myopathies are defined by descriptive names such as “spaghetti meat” (SM), “woody breast” (WB), and “white striping” (WS) [1, 2]. Spaghetti meat is characterized by the unraveling and splitting of muscle fibers; WB is characterized by increased consistency (compared to normal), pale color, and occasional petechial hemorrhages with variable exudate on the epimysial surface; and WS displays multiple white lines running parallel to the myofibers [3–5]. These myopathies are not caused by infectious agents, and therefore are not a public health concern [2]. However, the altered aspect of severely affected fillets can negatively influence consumers’ acceptance [6], leading to rejection or depreciation of fresh meat due to poor quality. Moreover, these myopathies have been associated with decreased nutritional and organoleptic qualities, such as higher fat and lower protein content compared to normal fillets [3, 7–9]. Meat processability issues, such as poor texture, higher drip and cooking losses as well as lower marinade uptake, have also been associated with these myopathies [10–12]. The negative impact of breast fillet myopathies has been estimated to be > $1 billion per year in North America alone [13].

Although it is clear that SM, WB, and WS are common in intensive broiler production systems, the reported prevalence of these myopathies varies between studies and countries. Zampiga et al. [14] in Italy reported a 29% and 6% prevalence of moderate and severe SM, respectively, in broiler with a live weigh of 2.9 kg at slaughter. Sihvo et al. [4] in Finland reported a 53% and 12% prevalence of moderate and severe WB, respectively, in broiler flock with an average live weight of 3.6 kg. Studies from France, Italy, Thailand, and the USA indicate that the prevalence of mild and moderate WS prevalence ranged from 36% to 96%, while severe WS ranged from 5% to 30% [14–18]. These myopathies often occur together, leading to a combination of myopathies with different severities [4, 5, 19].

Breast fillets affected by myopathies share overlapping histological changes, which are typically characterized by polyphasic degeneration and necrosis of myofibers, accumulation of inflammatory cells in the endomysium, and increased amounts of fibrous tissue and fat [4, 5, 20]. These histological changes are non-specific [4, 5], and offer limited information to discriminate between SM, WB, and WS.

Previous studies suggested that a multifactorial combination of genetic, nutritional, environmental, and management variables affect the development of breast myopathies [19, 21]. While breast muscle myopathies are observed more frequently in fast-growing, heavy broiler chickens [22, 23], an exact causative mechanism between body weight and myopathies remains elusive [1, 24, 25]. Bailey et al. [19] reported a relatively low heritability for these conditions (h^2^ = 0.04 for SM; h^2^ < 0.1 for WB; h^2^ < 0.338 for WS), emphasizing the importance of non-genetic factors, such as environment and management. On the other hand, an important genetic component has been suggested by other studies. For instance, Alnahhas et al. [18] documented a strong heritability (h^2^ = 0.65) of WS, and Livingston et al. [26] showed that the Ross 708 strain was more susceptible to higher severity of WB, when compared to the Cobb 500 strain raised under similar conditions. Feed formulation and dietary supplements have also been reported to affect the occurrence and severity of breast myopathies [4, 14, 15, 27–30]. Other growth parameters reported as potential predictors of WB and WS include breast fillet weight and yield [3, 31], average live weight at slaughter, and *P*. *major* length/width/depth [32].

While the prevalence of these myopathies has been documented in other countries with intensive poultry production, no such data are available for the Canadian poultry industry. Thus, the objectives of this study were to assess the prevalence of myopathies in commercial broiler chicken flocks from Ontario, Canada, and to investigate the risk factors associated with their occurrence. This information can be used to benchmark the status of myopathies in Canadian broilers, assess the magnitude of resulting economic losses, and enable future studies investigating the efficacy of mitigating strategies.

## Material & methods

### Sample size calculation and sample selection

The sample size to estimate the prevalence of myopathies was calculated based on our preliminary observations and using Eq (1). This was done by assuming a confidence level with 95% CI, an expected prevalence of at least 20% for each myopathy, and a 5% precision.

$$n=\frac{Z^{2}P(1-P)}{d^{2}}$$ Eq (1)

Where *n* = sample size; *Z* = *Z* statistic for a level of confidence; *P* = expected prevalence; *d* = precision [33].

The calculated sample size was 246 fillets per visit (37 visits in total), and four more samples were collected than the desired samples size from each flock to reduce the potential discrepancy from missing data. Therefore, 250 chicken fillets from each of 37 different broiler flocks (total, 9,250) were randomly selected and scored macroscopically at two processing plants in Ontario, Canada, between May 2019 and March 2020. One of every five fillets were sampled from the constantly running conveyor belt after deboning and chilling (approximately 105 min postmortem in air-chilling plant and 450 min postmortem in water-chilling plant), and placed back on the belt after scoring. No sorting of fillets was conducted at the plant before scoring by the investigative team.

### Macroscopic scoring

During each visit, the same two members of the investigative team visited each of the two slaughterhouses, and together scored each breast fillets to minimize scoring variations. In a few cases, when there were differing scores, the two scorers re-evaluated the fillets to reach an agreement. SM was categorized as either absent (SM0) or present (SM1) based on the presence of myofiber separation. WB and WS were scored using previously established criteria [4, 34], with some modifications. The severity for WB was divided into three categories, absent (WB0), moderate (WB1), and severe (WB2), based on firmness upon palpation of the breast fillets: WB0 had no firm areas; WB1 showed a consistency that was moderately firm in the cranial and/or caudal areas; WB2 showed a consistency that was markedly firm throughout the fillets. WS was classified into four categories, absent (WS0), mild (WS1), moderate (WS2), and severe (WS3), based on the number and thickness of white stripes running parallel to the muscle fibers (S1 Table). Normal fillets exhibited a cumulative score of SM0, WB0, and WS0. Co-occurrence of myopathies was recorded and visualized using an Excel spreadsheet and the VennDiagram (draw.triple.venn) package implemented in the R statistical software [35].

### Questionnaire design and coding of variables

A questionnaire (Table 1), devised to collect information on risk factors, was sent to both processing plants. De-identified data for each flock were provided by the processing plant personnel based on flock sheets from growers. The survey included a total of 24 questions, which focused on flock demographics (e.g., *age*, *average live weight*) and a list of parameters concerning the farm, flock health, transportation conditions, and variables directly related to operations at the processing plants.

**Table 1:** Summary of data collected from the questionnaire and Environment Canada weather stations for the analysis of the occurrence of breast myopathies.

*Variables obtained from questionnaire to processing plants (24 questions)*
**Farm variables**
• Feed type (categorical)^†^
•Lighting program (categorical) ^†^
• Number of floors in a barn (1 or >2, categorical)
• Forward sortation area (FSA) of growers and hatcheries (categorical) ^‡^
**Flock demographics variables**
• Age (d, continuous)
• Average live weight at slaughter (g, continuous)
•Breed (categorical) ^†^
• Sex (categorical)
• Sources of chicks (categorical)
**Flock health variables**
• Disease observed (categorical)
• Mortality rate during grow-out (%, continuous)
• Raised without antibiotics (categorical)
• Vaccination and medication records (categorical)
**Transportation from farms to processing plant variables**
• Birds per crate (b/c, continuous)
• Dead-on arrival (%, continuous)
• Feed withdrawal time*
• Water withdrawal time*
• Time of load start*
• Time of load end*
• Truck travel time (min, continuous)
**Processing plant variables**
• Hold time on lairage at plant (min, continuous)
• Time of exsanguination*
•Exsanguination to deboning time (min, continuous) ^†^
• Condemnation (%, continuous)
*Variables obtained from Environment Canada weather stations*
**Environmental variables**
•Average temperature during grow-out and transport (°C, continuous)
• Precipitation during grow-out and transport (mm, continuous)
• Season (categorical)

* The processing plants provided the times of each event, and these times were used to calculate the duration of specific segments (S1 Fig).

^†^ Variables were excluded from further analyses because of lack of variability.

^‡^ Questions on egg storage time and incubation temperature were asked but not answered.

In the questionnaire, *mortality rate during grow-out* (%) was defined as the proportion of dead birds during the entire growing period in the barn: mortality rate during grow-out (%) = (number of chicks placed–number of birds being shipped) × 100 / number of chicks placed. *Dead on arrival* (*DOA*, %) was defined as the number of dead birds on arrival at the processing plant divided by the number of live birds loaded per trailer. *Disease observed* was defined as disease outbreaks including those necessitating a veterinary treatment during the grow-out [36]. *Condemnation* (%) was defined as the number of birds condemned during inspection at the processing plant divided by the number of birds loaded in each trailer [37].

Dichotomous categorical variables (i.e., raised without antibiotics (*RWA*) and *lighting program* [only two programs were used])] in the questionnaire were coded as true/false (i.e., 1/0). Non-dichotomous categorical variables were coded using nominal variables. The time of the year during which fillets were sampled was divided into *season*: spring (20 Mar 2019–20 Jun 2019), summer (21 Jun 2019–22 Sep 2019), fall (23 Sep 2019–21 Dec 2019), and winter (22 Dec 2019–19 Mar 2020) [38]. The *sex* of the flock was categorized as male, female or mixed, and the *source of chicks* was categorized as Canadian domestic, from the USA, or mixed (for birds derived from both; no other sources were identified). Open questions included *vaccination* and *medication records*, and *diseases* experienced by the flock. Reported medications were grouped into 4 antimicrobials classes (I–IV), according to Health Canada’s classification [39]. Ionophores (category IV antimicrobials) and chemical coccidiostats were grouped together. Reported vaccination were nominally classified based on the pathogen, including vaccination against coccidia, *E*.*coli*, infectious bursal disease virus, Marek’s disease virus, and avian reovirus (i.e., infectious tenosynovitis) [40]. After nominal classification, the categories were further dichotomized into 1 and 0, indicating use or no-use of the medication or vaccine during the flock’s life. Reported flock diseases were dichotomized into 0 (denoting that flocks did not have a disease) and 1 (denoting that the flocks had a disease). Lastly, farm location was assigned by the first three characters of the Canadian postal code (forward sortation area, *FSA*) (Table 1).

From the data collected in the questionnaire, a set of four secondary environmental information was derived from Environment Canada, based on weather stations [41] (Table 1). Variables related to farm location included the *mean temperature* (°C) and *total precipitation* (mm) during the grow-out period. When there was one weather station in an FSA, the temperature and precipitation values from that weather station were collected. When there were more than two weather stations in an FSA, there average values were used. When there were no weather stations in an FSA, the weather station closest to the FSA centroid was selected, using the ‘measure distance’ function from Google Maps. The temperature and precipitation during the grow-out period were determined by the mean temperature and total precipitation for the month encompassing the longer portion of the grow-out period (e.g., when birds were raised from Jan 1^st^ to Feb 11^th^, values during January were selected). The *temperature* and *precipitation during transport* variables indicated the mean temperature (°C) and total precipitation (mm) on the day of shipping from the farm.

For each flock, the processing plants provided the times of feed and water withdrawal, load start and end, truck arrival time to the processing plant, and start of exsanguination. These times were used to calculate three additional variables, including *the duration without feed*, *duration without water*, *and loading duration* (Table 1 and S1 Fig). Data derived either directly from the questionnaire, or secondarily through additional calculation or environmental assessment, were considered as possible risk factors, and were used in exploratory statistics.

### Statistical analyses

#### Descriptive statistics

A five-number summary (i.e., values representing the minimum and maximum, the lower and upper quartiles, and the median) was computed for the continuous variables, and frequencies were calculated for the categorical variables. Differences in the frequency of SM and WB across seasons were compared using the Kruskal-Wallis test, and Dunn’s multiple range test was used for post-hoc multiple comparisons.

#### Exploratory statistics

Multivariable regression analysis was conducted to estimate the association between risk factors (independent variables) and presence/absence of SM or WB (outcome variables), considered separately (i.e., one model for each myopathy) and regardless of severity. Hence, WB was re-coded as 0 (denoting WB0) and 1 (denoting WB1 and WB2) for the logistic regression analyses. WS was not considered for regression analysis because WS1 was present in 93.8% of fillets.

Risk factors to be fitted in the final multivariable model were initially tested individually (univariable models) using binomial logistic regression due to the binary nature of the dependent variables (presence/absence), and employing a relaxed significant threshold (p < 0.02). When continuous variables were significant from univariable models, they were kept in the model as continuous variables. If they were not significant during unconditional association analysis, they were categorized into three equal quartiles (low, medium, high) using the *xtile* function from a statistical software package (Stata 14.0; Stata Corporation, College Station, Texas, USA), and tested again as categorical categories using univariable models. These variables included *average live weight*, *precipitation during grow-out*, *precipitation during transport*, *temperature during grow-out*, *temperature during transport*, *mortality rate during grow-out*, *hold time on lairage at plant*, *and condemnation*.

Pair-wise correlation coefficients, using Spearman’s rank test among all significant independent variables, were examined to prevent the inclusion of collinear variables in the multivariable models. When two variables were highly correlated (rho > 0.70; p < 0.05), the one with the smallest p-value was considered for inclusion in the multivariable model. All unconditionally significant variables identified during univariable screening were offered to a multivariable model, and a manual backward elimination process was performed. Variables with p > 0.05, as calculated using the likelihood ratio test, were removed for a better fit of a model. For suspected confounders, when the removal of a variable changed the coefficients of the remaining variables from the final model by more than 20%, the variable was kept in the final model, otherwise, it was dropped [42].

After the final independent variables were selected (S2 Fig), in order to evaluate and control for clustering in the occurrence of breast myopathies, 2-level random intercept logistic regression models were built [43]. Flocks were included in the models as random effects. Based on the unexplained variance components at each level of the models (breast fillets and flocks), intra-class correlation coefficients (ICC) were calculated with flocks as the level-2 variable. The ICC was computed by assuming that level 1 variance on the logit scale was:

$$\pi^{2}÷3=3.29,\;\pi =3.1416$$ Eq 2

The formula used for the ICC calculation for the 2-level model was,

$$\mathrm{ICC}=\frac{\sigma^{2}(flock)}{\sigma^{2}(flock)+3.29}$$ Eq 3

The final results of the random intercept 2-level (flock-fillet) multivariable logistic regression models (one for SM, another for WB) were reported as the odds ratio (95% CI) of breast myopathies to occur, as an effect of the independent variable(s) included in the model (p < 0.05). An odds ratio > 1 indicates increased odds of a certain myopathy to occur, as a result of the predictor variable to increase by one unit (continuous) or in comparison to a set reference (categorical), whereas the odds ratio < 1 denotes a decreased chance.

## Results

### Data description

A total of 9,250 fillets from 37 commercial broiler flocks were scored for the presence and severity of breast myopathies; 4,750 fillets from 19 flocks (5 from spring 2019, 5 from summer 2019, 5 from fall 2019, and 4 from winter 2020) were scored at a plant employing air-chilling (Plant A). The other 4,500 fillets from 18 flocks (5 from summer 2019, 6 from fall 2019, and 7 from winter 2019) were scored at a plant employing water-chilling (Plant B). The visit for spring 2020 was not possible due to COVID-19-related restrictions. Plant A, which was visited during all four seasons, was selected to evaluate the seasonal effect on breast myopathy occurrence.

After primary and derived questionnaire data were formatted, a total of 15 continuous and 18 categorical variables were defined as possible predictors of myopathy development for the regression models. Table 2 shows the five-number summary of continuous variables, and Table 3 shows the proportion of categorical variables. All 37 flocks were Ross 708 and fed with corn-based rations, so the *breed* and *feed* variables were excluded from the regression analyses. Infectious bronchitis and Marek’s disease vaccine were administered in 100% and 92% of flocks, respectively, whereas *E*. *coli* vaccine and infectious tenosynovitis vaccine were administered to less than 20% of the flocks. Due to the high frequency in the dataset, the *bronchitis vaccine* variable was excluded from further analysis. Only 2 lighting programs were used, one employed by all RWA growers, and one by the rest. Thus, it was not necessary to include the *lighting program* variable in the regression analysis.

**Table 2:** Descriptive statistics of 15 continuous variables from 37 chicken broiler flocks surveyed in a study on the occurrence of breast meat myopathies in Ontario, Canada (2019–2020).

Variable	Minimum		Q1	Median	Q3	Maximum
*Continuous variables obtained from questionnaire to processing plants*						
**Flock demographics variables**						
Age (d)	36		39	40	42	43
Average live weight at slaughter (g)	1,940		2,260	2,397	2,490	2,630
**Flock health variables**						
Mortality rate during grow-out (%)	1.25		2	2.33	3	5.8
**Transportation from farms to processing plant factors**						
Birds per crate (b/c)	8		9	9	10.5	12
Loading duration (min)	40		70	90	105	150
Dead-on arrival (%)	0		0.03	0.06	0.09	0.16
Duration without feed (min)	300		616	705	792	1005
Duration without water (min)	113		202	318	371	470
Truck travel time (min)	20		57	120	199	798
**Processing plant variables**						
Hold time on lairage at plant (min)	10		98	130	174	269
Condemnation (%)	0.01		0.34	0.75	1.24	2.81
*Continuous variables obtained from Environment Canada weather station*						
**Environmental variables**						
Precipitation during grow-out (mm)		0	54	74.3	106	164.6
Temperature during grow-out (°C)		-9.8	-1.2	10.5	15.8	22.7
Precipitation during transport (mm)		0	0	0.4	2.1	20.2
Temperature during transport (°C)		-9.8	1	9.4	19.1	23.7

**Table 3:** Descriptive statistics of 18 categorical variables from 37 chicken broiler flocks surveyed in a study on the occurrence of breast myopathies in Ontario, Canada (2019–2020).

Variables	Number (%) of response	Variables	Number (%) of response
**Farm variables**		**Flock health variables-continued**	
Number of floors in barns		Bronchitis vaccine	
> 2 floors	25 (67.6)	Administered	37 (100)
1 floor	12 (32.4)	Not administered	0 (0)
FSA* of growers		Coccidiosis vaccine	
L0G	1 (2.7)	Administered	6 (16.2)
L0R	1 (2.7)	Not administered	31 (83.8)
L9H	1 (2.7)	*E*. *coli* vaccine	
N0B	2 (5.4)	Administered	2 (5.4)
N0G	8 (21.6)	Not administered	35 (94.6)
N0J	2 (5.4)	Infectious bursal disease vaccine	
N0K	4 (10.8)	Administered	32 (86.5)
N0L	3 (8.1)	Not administered	5 (13.5)
N0M	5 (13.5)	Infectious tenosynovitis vaccine	
N3B	2 (5.4)	Administered	7 (18.9)
N4G	1 (2.7)	Not administered	30 (81.1)
N4S	3 (8.1)	Marek’s disease vaccine	
N4W	1 (2.7)	Administered	34 (91.9)
N7A	3 (8.1)	Not administered	3 (8.1)
FSA of hatcheries		Category II antimicrobials^a^	
N0G	2 (5.4)	Administered	2 (5.4)
N4N	19 (51.4)	Not administered	35 (94.6)
N5A	16 (43.2)	Category III antimicrobials^b^	
**Flock demographics variables**		Administered	26 (67.6)
Sex of broilers		Not administered	12 (32.4)
Female	15 (40.5)	Coccidiostat and/or ionophores	
Male	11 (29.7)	Administered	27 (73.0)
Mixed	11 (29.7)	Not administered	10 (27.0)
Source of chicks		**Processing plant variable**	
Domestic	29 (78.4)	Chilling method	
Mixed	4 (10.8)	Air-chilling	19 (51.4)
USA	4 (10.8)	Water-chilling	18 (48.6)
**Flock health variables**		**Environmental variable**	
Disease observed		Season	
Observed	4 (10.8)	Spring	5 (13.5)
Not observed	33 (89.2)	Summer	10 (27.0)
Raised without antibiotics (RWA)		Fall	11 (29.7)
RWA flocks	8 (21.6)	Winter	11 (29.7)
Non-RWA flocks	29 (78.4)		

*Forward sortation area.

^a^Category II (High Importance) antimicrobials included amoxicillin and sulfadiazine, and trimethoprim.

^b^Category III (Medium Importance) antimicrobials included avilamycin, bacitracin, sulfaquinoxaline, and pyrimethamine.

Similarly, time between exsanguination and deboning were consistent within each of the plants (Plant A = 105 min; Plant B = 450 min), therefore we excluded the variable *exsanguination to deboning time* from the regression analysis. The *FSAs of growers* variable was excluded because data were widely dispersed resulting in substantial standard errors and p-values.

Moreover, independent categorical variables were excluded if they presented less than 5.5% in any given observation, in order to reduce small-sample bias in maximum likelihood estimation. To avoid small observation values, locations of the hatcheries (FSA) were combined to create the new variable *North*, which combined N0G (5.4%) with N4N (51.4%), while the variable *South* variable included the remaining N5A (43.2%).

In Canada, growers can claim RWA flock when broilers are raised with chemical coccidiostats, but not ionophores [44]. Regarding the non-RWA flocks in our cohort, 26 received coccidiostats and/or ionophores, and three flocks received category II antimicrobials (e.g., amoxicillin) and/or category III antimicrobials (e.g., bacitracin) but not coccidiostats and/or ionophores. The 8 RWA flocks did not receive any antimicrobial, including coccidiostats or ionophores. Since RWA flocks and coccidiostats/ionophores usage were strongly correlated (rho = 0.76), the *coccidiostats and/or ionophores* variable was excluded during correlation analysis. On the contrary, the correlation coefficient between *coccidiosis vaccine* and *RWA* was 0.66, and therefore both variables were kept in final model.

### Prevalence

**Fig 1:**
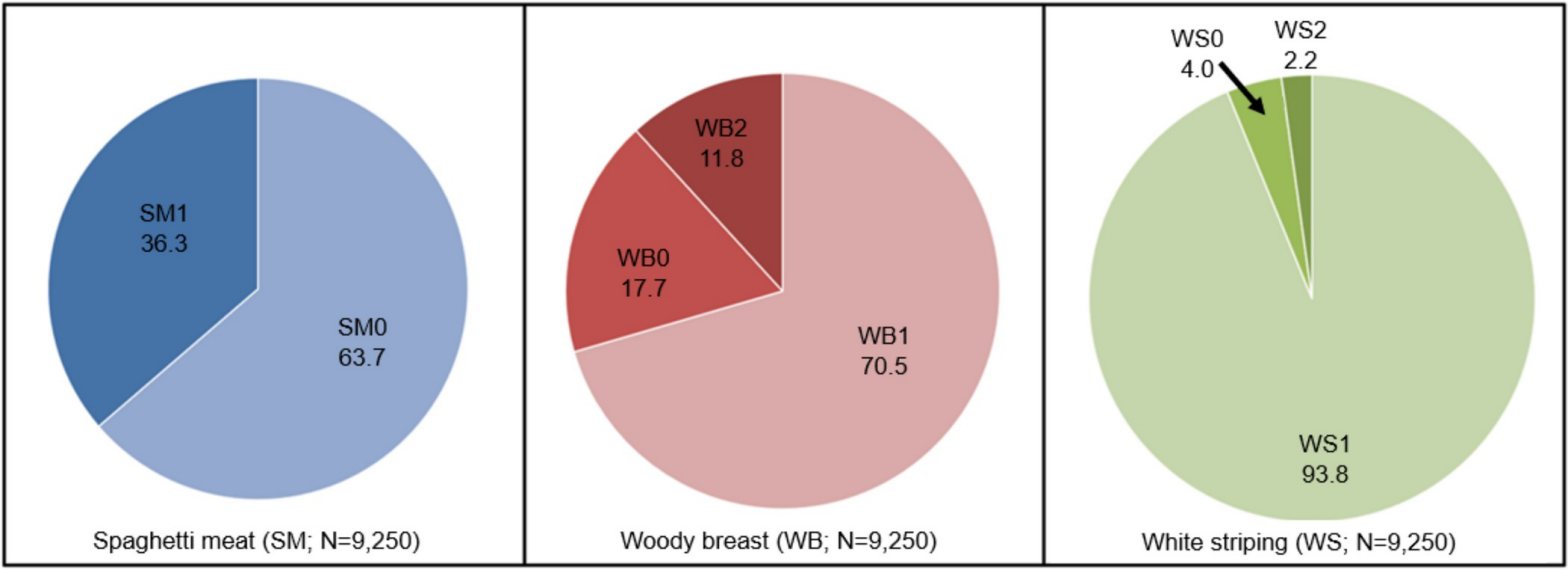
Pie charts illustrating the prevalence of spaghetti meat (SM), woody breast (WB), and white striping (WS). The prevalence of SM (SM1) was 36.3%. The prevalence of moderate WB (WB1) was 70.5%, and the prevalence of severe WB (WB2) was 11.8%. The prevalence of mild WS (WS1) was 93.8%, and the prevalence of moderate WS (WS2) was 2.2%. There was no severe WS (WS3) observed in our samples (n = 9,250; average live weight at slaughter: 2.36 kg).

**Fig 2:**
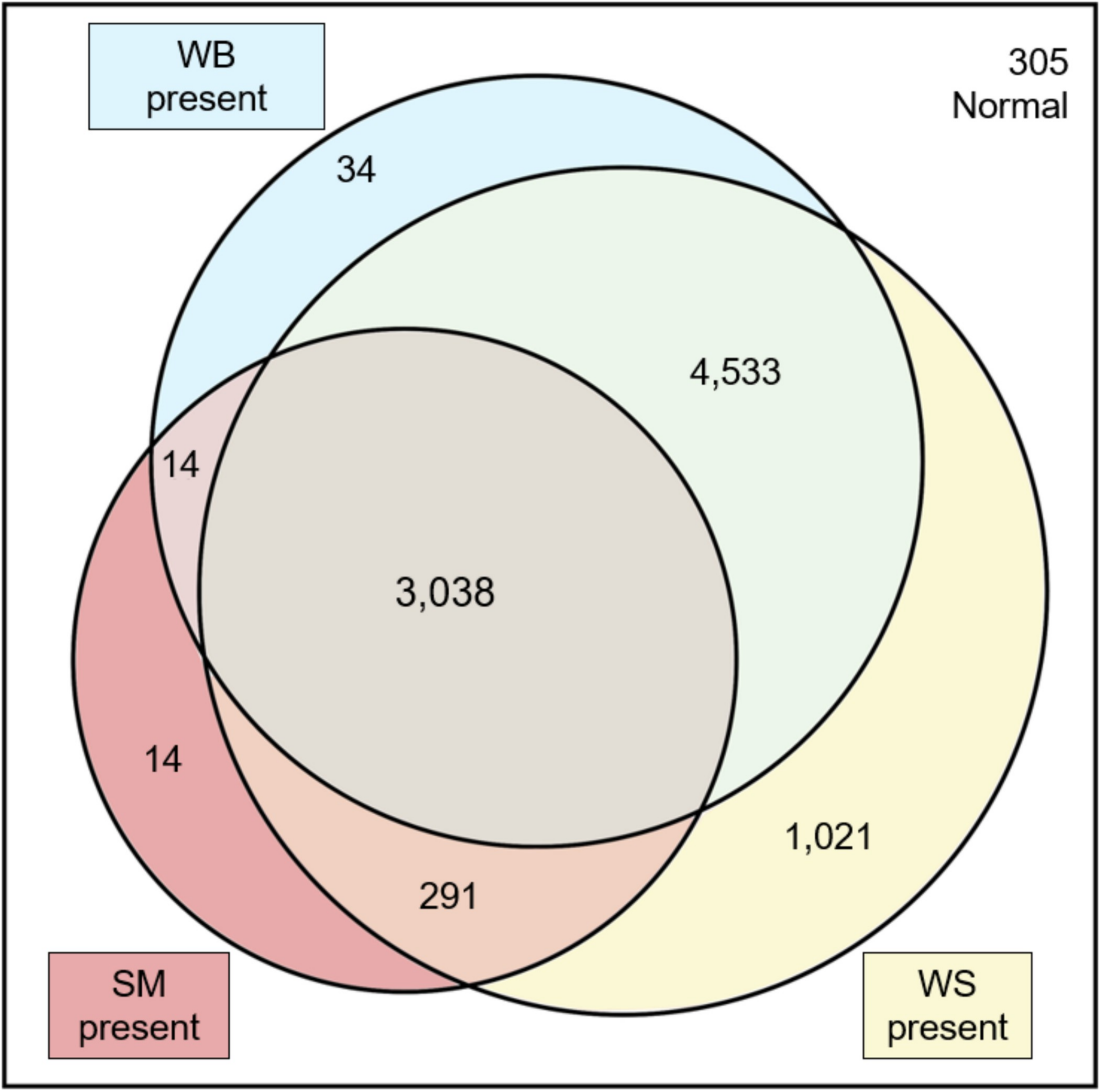
Venn diagram illustrating the incidence counts of spaghetti meat (SM), woody breast (WB), and white striping (WS) in 9,250 fillets. A total of 7,876 fillets presented multiple myopathies together. Within 7,876 fillets, 3,038 fillets presented SM and WB and WS together. Out of 9,250 scored fillets, only 305 fillets did not show myopathies. SM0: SM absent, SM1: SM present. WB0: WB absent, WB1: WB moderate, WB2: WB severe. WS0: WS absent, WS1: WS mild, WS2: WS moderate.

Fig 1 summarizes the prevalence of myopathies in 9,250 broiler chicken fillets examined. The overall prevalence of SM1, total WB (i.e., WB1 plus WB2), and total WS (i.e., WS1 plus WS2), regardless of severity or co-occurrence, was 36.3% (95% CI 35.3–37.3), 82.4% (95% CI 81.6–83.1), and 96.0% (95% CI 95.6–96.4), respectively. In our cohort, no WS3 was observed. Only 3.3% (95% CI 2.9–3.7) of fillets showed no myopathies (i.e., SM0, WB0, and SM0). The prevalence of WB1 was 70.5% (95% CI 69.6–71.5), and the prevalence of WB2 was 11.8% (95% CI 11.2–12.5). The prevalence of WS1 was 93.8% (95% CI 93.4–94.4), and the prevalence of WS2 was 2.2% (95% CI 1.9–2.5). The range of SM1 prevalence varied from 10.4% to 66.8%, and WB2 ranged from 2.4% to 32.8% across flocks. Almost all fillets (96.7%) presented at least one myopathy, and numerous fillets presented multiple myopathies together (85.1%). Moreover, 36.1% of fillets presented both SM and WB, and 32.8% of fillets presented SM and WB and WS together (Fig 2).

**Fig 3:**
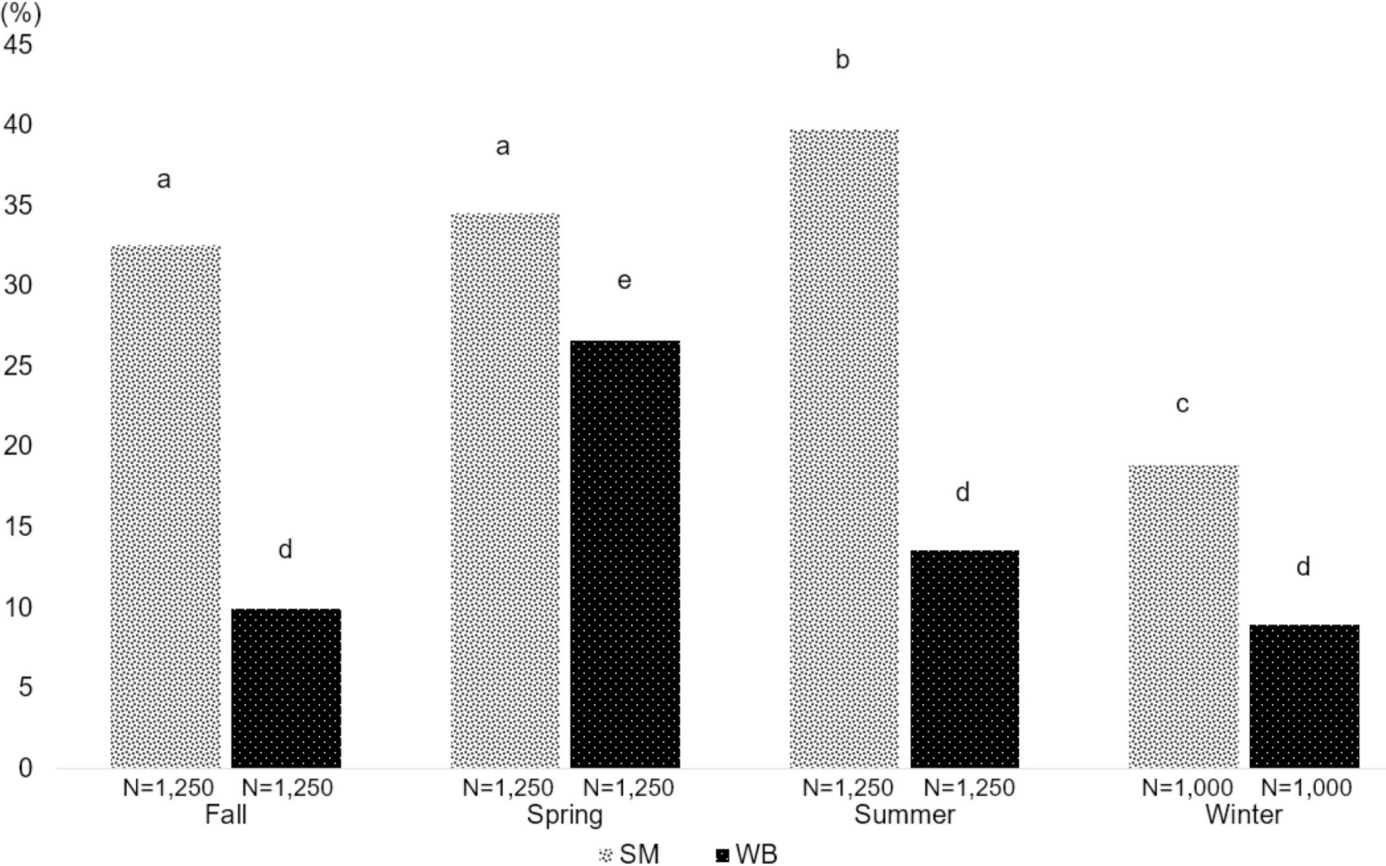
Seasonal variations in the prevalence of spaghetti meat (SM) and severe woody breast (WB2). A total of 4,750 fillets (19 flocks) were scored at a processing plant that employs air-chilling. The prevalence of SM1 was the highest during the summer (39.8%), whereas the prevalence of WB2 was the highest during spring (26.6%). Different letters above bars indicate significant differences among groups (Kruskal Wallis test with Dunn’s test for multiple comparisons, p < 0.05) of SM, and WB in each season.

The prevalence of SM was significantly higher in the processing plant adopting the water-chilling (39.8%, 95% CI: 38.4 to 41.3) compared to the air-chilling (32.1%, 95% CI: 30.8 to 33.4) method (t-test; p < 0.001, n = 9,250). To assess seasonal variability, the prevalence of SM1 and WB2 was assessed in 4,750 fillets processed at Plant A, for which observations from an entire year were available. There were statistical differences (Kruskal Wallis test with Dunn’s test for multiple comparisons) in the prevalence of SM1 and WB2 between seasons (Fig 3). The SM1 prevalence was the highest during summer (39.8%, 95% CI 38.4–41.2), and the lowest during winter (18.9%, 95% CI 17.8–20.1). Meanwhile, the WB2 prevalence was the highest during spring (26.6%, 95% CI 25.4–27.9), and the lowest during winter (9.0%, 95% CI 8.2–9.9).

### Univariable model for SM occurrence

Unconditional associations between the SM occurrence, regardless of severity, and all the risk factors are presented as supporting information (S2 Table). To build a multivariable logistic regression model for the occurrence of SM, information on 36 variables was collected from processing plants and weather stations. Seven variables with low variability (< 5.5%) were removed. Twenty-nine variables were used for unconditional associations, and 5 variables were removed using a relaxed p-value (p < 0.2). Twenty-four variables were used for the correlation analysis, and 4 variables were removed because of strong collinearity (rho > 0.7). Multivariable logistic regression models were built for 20 variables. Thirteen variables were removed using the likelihood ratio test because removing variables result in better fits of the model. The final model included 7 variables (S2A Fig).

### Final multivariable model for SM occurrence

The results of the final random intercept 2-level (flock-fillet) multivariable logistic model for the SM occurrence are presented in Table 4.

**Table 4:** Results of the final random intercept 2-level (flock-fillet) multivariable logistic regression model for spaghetti meat, in a cohort of 9,250 chicken breast fillets collected at two processing plants between 2019–2020 in Ontario, Canada.

	Variable	Value	Odds Ratio	P value	95% CI
Flock demographics	Average live weight (g)	Low (1940–2290)	Reference		
		Medium (2300–2450)	1.13	0.352	0.87–1.47
		High (2460–2630)	1.30	0.044	1.01–1.69
	Source of chicks	Domestic	Reference		
		Mixed	0.91	0.610	0.63–1.31
		USA	1.50	0.043	1.01–2.23
Flock health	Coccidiosis vaccine	Administered	1.46	0.018	1.07–2.00
		Not administered	Reference		
	Infectious bursal disease vaccine	Administered	1.84	< 0.001	1.32–2.54
		Not administered	Reference		
Transportation	Truck travel time (min)	Low (20–68)	1.64	0.002	1.21–2.22
		Medium (69–163)	1.89	< 0.001	1.43–2.49
		High (164–798)	Reference		
Processing plant	Hold time on lairage at plant (min)	Low (10–110)	1.26	0.092	0.96–1.64
		Medium (111–160)	1.35	0.038	1.02–1.80
		High (161–269)	Reference		
Environmental	Temperature during grow-out (°C)	Low (-9.8–0)	Reference		
		Medium (0.1–15.7)	2.58	< 0.001	1.96–3.39
		High (15.8–22.7)	1.75	< 0.001	1.31–2.34

#### Flock demographics variables

The occurrence of SM was associated with the *average live weight* of broilers or *source of chicks*. When broilers were heavier than 2.45 kg at slaughter, they had higher odds of having SM compared with those smaller than 2.30 kg (OR = 1.30, 95% CI 1.01–1.69). Day-old chicks and/or hatching eggs sourced from the USA had significantly higher odds of SM occurrence than domestic-only flocks (OR = 1.50, 95% CI 1.01–2.23).

#### Flock health variables

Vaccination for coccidiosis and infectious bursal disease was associated with the occurrence of SM. Flocks that received the *coccidiosis vaccine* had significantly higher odds of SM occurrence than flocks that did not receive the vaccine (OR = 1.46, 95% CI 1.07–2.00). Similarly, flocks that received the *infectious bursal disease vaccine* had significantly higher odds of SM occurrence than flocks that did not receive the vaccine (OR = 1.84, 95% CI 1.32–2.54).

#### Transportation variables

When *truck travel time* was less than 69 min, broilers had higher odds of having SM compared with those that travelled 164 min or more (OR = 1.64, 95% CI 1.21–2.22). Likewise, when *truck travel time* was equal to or more than 69 min and less than 164, broilers had higher odds of having SM compared with those that travelled for 164 min or more (OR = 1.89, 95% CI 1.43–2.49).

#### Processing plant variables

When *hold time on lairage at plant* was equal to or more than 111 min and less than 161, broilers had higher odds of having SM compared with those that were held for 161 min or more (OR = 1.35, 95% CI 1.02–1.80).

#### Environmental variables

*The temperature during grow-out* was associated with the occurrence of SM. When *temperature during grow-out* was higher than 0°C and lower than 15.8 °C, broilers had higher odds of having SM compared to those flocks for which temperature was equal to or lower than 0°C (OR = 2.58, 95% CI 1.96–3.39). When *temperature during grow-out* was equal to or higher than 15.8°C, broilers had higher odds of having SM compared to those flocks for which temperature was equal to or lower than 0°C (OR = 1.75, 95% CI 1.31–2.34).

### Univariable model for WB occurrence

Unconditional associations between the occurrence of the breast myopathy (WB) and all factors of interest are presented as supporting information (S2 Table). To build a multivariable logistic regression model for the occurrence of WB, information on 36 variables was collected from processing plants and weather stations. Seven variables with low variability (< 5.5%) were removed. Twenty-nine variables were used for unconditional associations, and ten variables were removed using a relaxed p-value (p < 0.2). Nineteen variables were used for the correlation analysis, and 3 variables were removed because of strong collinearity (rho > 0.7). Multivariable logistic regression models were built for 16 variables. Seven variables were removed using the likelihood ratio test for a better fit of the model. The final model included 9 variables (S2B Fig).

### Final multivariable model for WB occurrence

The results of the final random intercept 2-level (flock-fillet) multivariable logistic model for the WB occurrence are presented in Table 5.

**Table 5:** Results of the final random intercept 2-level (flock-fillet) multivariable regression model for woody breast, in a cohort of 9,250 chicken breast fillets collected at two processing plants between 2019–2020 in Ontario, Canada (n = 9,250 fillets).

	Variable	Value	Odds Ratio	P value		95% CI
Flock demographics	Average live weight (g)	Low (1,940–2,290)	Reference			
		Medium (2,300–2,450)	1.18	0.042		1.01–1.38
		High (2,460–2,360)	1.23	0.020		1.03–1.47
	Source of chicks	Domestic	1.32	0.028		1.03–1.69
		Mixed	1.10	0.574		0.79–1.54
		USA	Reference			
Flock health	Mortality rate during grow-out (%)	Low (1.25–2.0)	1.79	<0.001		1.43–2.25
		Medium (2.1–3.0)	1.76	<0.001		1.42–2.17
		High (3.1–5.8)	Reference			
	Coccidiosis vaccine	Administered	Reference			
		Not administered	1.86	<0.001		1.51–2.29
Transportation	Birds per crate	Low (8–9)	Reference			
		Medium (9.5–10)	0.83	0.199		0.62–1.10
		High (10.5–12)	1.46	<0.001		1.18–1.79
	Dead on arrival (%)	0–0.16	1.04	<0.001		1.02–1.05
	Loading duration (min)	Low (40–75)	Reference			
		Medium (76–100)	1.19	0.111		0.96–1.47
		High (101–150)	1.48	0.001		1.18–1.86
Processing plant	Hold time on lairage at plant (min)	Low (10–110)	1.31	0.014		1.06–1.61
		Medium (111–160)	Reference			
		High (161–269)	1.52	<0.001		1.24–1.86
Environmental	Season	Spring	1.06	0.782		0.65–1.71
		Summer	1.33	0.020		1.05–1.70
		Fall	1.19	0.436		0.79–1.82
		Winter	Reference			

#### Flock demographics variables

The occurrence of WB was associated with the *average live weight* at slaughter or *source of chicks*. When broilers were heavier than 2.29 kg and smaller than 2.46 kg, they had higher odds of having WB compared with those smaller than 2.30 kg (OR = 1.18, 95% CI 1.01–1.38). When broilers were heavier than 2.45 kg, they had higher odds of having WB compared with those smaller than 2.30 kg (OR = 1.23, 95% CI 1.03–1.47). Day-old chicks and/or hatching eggs sourced domestically had significantly higher odds of WB occurrence than USA-only flocks (OR = 1.32, 95% CI 1.03–1.69).

#### Flock health variables

*Mortality rate during grow-out* and *coccidiosis vaccine* were associated with the occurrence of WB. When the mortality rate during grow-out was lower than 2.1%, broilers had higher odds of having WB compared with those higher than 3.0% (OR = 1.79, 95% CI 1.43–2.25). When the mortality rate during grow-out was higher than 2.0% and lower than 3.1%, broilers had higher odds of having WB compared with flock mortality higher than 3.0% (OR = 1.76, 95% CI 1.42–2.17). Flocks that did not receive the coccidiosis vaccine had a significantly higher risk of the WB occurrence than flocks that receive the vaccine (OR = 1.86, 95% CI 1.51–2.29).

#### Transportation variables

*Birds per crate*, *dead on arrival*, and *loading duration* were associated with the occurrence of WB. When more than 10 birds were in a crate, broilers had higher odds of having WB compared with those equal to or less than 9 birds (OR = 1.46, 95% CI 1.18–1.79). For every one-unit (%) increase in DOA, a 1.04 increase in the odds of WB development was expected. When *loading duration* was longer than 100 min, broilers had higher odds of having WB compared with those loaded for less than 76 min (OR = 1.48, 95% CI 1.18–1.86).

#### Processing plant and environmental variables

*Hold time on lairage at plant* was associated with the occurrence of WB. When broilers were held on lairage less than 111 min, they had higher odds of having WB compared with those held longer than 110 min or less than 161 min (OR = 1.31, 95% CI 1.06–1.61). When broilers were held on lairage longer than 160 min, they had higher odds of having WB compared with those held longer than 110 min or less than 161 min (OR = 1.52, 95% CI 1.24–1.86). The *season* affected the occurrence of WB. During summer, a 2.38 increase in the odds of being WB was expected compared to winter.

### Random effects of flocks

The final random-intercept 2-level models showed significant variances at the flock level. The flock variance of SM and WB, as calculated in the final models, was 0.06 and 0.01, respectively. The ICC of SM between flocks was 0.018 indicating that 1.8% of the total variation in the SM occurrence was accounted for by the flock, leaving 98.2% of the variation accounted for by the fillets. Similarly, the ICC of WB between flocks was 0.003, indicating that 0.3% of the total variation in the WB occurrence was accounted for by the flock, leaving 99.7% of the variation was accounted for by the fillets.

## Discussion

### Prevalence of breast myopathies

The present study shows that SM, WB, and WS occur in Ontario flocks with a high prevalence. The first myopathy reported in the literature, WS, was observed in 96.0% of scored fillets in our cohort, although fillets moderately affected by WS (WS2) accounted for only 2.2%, and no severe WS (WS3) was detected. Our findings are in partial agreement with previous studies, which reported the prevalence of WS, regardless of severity, to range between 43.0% and 96.0%. In those studies, WS2 constituted between 26.5% and 38.8% of total fillets evaluated [3, 14, 16, 17, 22, 45], and WS3 ranged between 3.3% and 15.2% [18, 45]. The lower prevalence of moderate and severe WS in our cohort may have been the result of a lower average live age and weight at slaughter. In our study, age and weight ranges were 36–43 d and 1.9–2.6 kg, while in the aforementioned reports, age ranged between 42 to 54 days and weight varied from 2.3 kg to 3.8 kg. This is in agreement with the general consensus, indicating that myopathies tend to develop more frequently in heavier and older birds [2, 23]. Furthermore, the subjective classification criteria for these myopathies may affect our ability to classify moderate and severe WS, warranting caution in making direct comparisons between studies.

The prevalence of WB in our study was 82.4%, with fillets with a severe form (WB2) accounting for 11.9% of the total. Other studies reported similar prevalence estimates, with total WB ranging from 64.8% to 90.8%, and WB2 affecting between 11.9% to 47.9% of fillets [4, 14, 18, 28]. Some degrees of fluctuation between studies are likely due to different ages, sex, and the average live weights at slaughter.

The prevalence of SM in the flocks examined was 36.3%, which is slightly higher (35%) than what was reported in Italy [14]. In that study, SM was evaluated using a pinching method (to check for the tendency of myofiber separation) with a three-tier classification system, showing that the prevalence of mild SM was 29% and severe SM was 6%. In our study, SM was assessed based on a visual evaluation only after the chilling process, which already implies manipulation (e.g., massaging) of the fillets. This mechanical effect may have contributed to myofiber separation, possibly facilitating detection of SM and leading to somewhat higher frequency.

We observed a relatively high frequency (85.1%) of breast fillets which present at least two myopathies. This finding is consistent with that of other studies, which reported co-occurrence of WB and WS in particular [4, 5, 28], and suggests that pectoral muscle myopathies may have some underlying causes in common [1], as indicated also by overlapping histological changes of the affected pectoral muscles [4, 5, 18].

Overall, our study shows that myopathies with a potential to affect consumer perception of meat quality (SM1 and WB2) account for approximately 10% to 30% of fillets, while milder forms of myopathies (WB1 and WS1) are well over 50%. These figures have the capacity to severely impact Canadian producers, meat processors, and retailers. It has been documented that consumer acceptance of breast fillets affected by WS is lower than that of normal fillets, due to a fatty appearance of the white-striped breast fillets [6], and that WS result in low consumer acceptability and purchase intent [46]. Similarly, WB affects sensory characteristics and can negatively impact consumer preference [47].

### Risk factors

Little research has been done on risk factors related to the occurrence of breast myopathies. Our analysis has shown that the occurrence of SM and WB breast myopathies in Ontario broilers is affected by multiple risk factors associated with the flock demographics and source of birds, flock health, transportation, processing plant, and environmental factors. The discussion below contextualizes the results of the multivariable regression analysis based on these main areas.

#### Flock demographics variables

Our model shows that the odds of SM and WB increase when the *average live weight* at slaughter increases, i.e., the odds of SM and WB were increased when the average live weight was ≥ 2460g and 2300g, respectively. The positive association between WB and heavier birds is supported by numerous studies, and it has been proposed that the increased number, diameter, and length of muscle fibers, as seen in heavier broilers, can result in reduced blood vessel density, eventually leading to muscle fibers loss and replacement with connective tissue [48, 49]. To the authors’ knowledge, however, this is the first study documenting an association between SM and heavier birds, although the mechanism underpinning this association remains unclear. Reducing the growth rate and/or the final bodyweight of the birds would likely be able to reduce the occurrence of SM and WB; however, these are trade-offs with expected yield and performance consequences.

In Canada, approximately 78.9% of hatching eggs and chicks are domestically sourced, and 21.1% of broiler hatching eggs and chicks (hatching eggs 17.4%, chicks 3.7%) are imported from the USA under the regulations of the United States-Mexico-Canada Agreement (USMCA) and the World Trade Organization (WTO) [50, 51]. In our dataset, the domestic-only flocks accounted for 78.4%, which is similar to the proportion mandated by the USMCA and WTO agreements. In our cohort, the *source of chicks* affected the occurrence of SM and WB, with flocks that were entirely or in part domestically sourced showing higher odds of WB. It is unclear how the *source of chicks* could directly affect development of myopathies, however this parameter likely includes variability at the hatchery and parent flock level, as well as different transportation conditions of hatching eggs and chicks. Variables associated with breeder flocks can include age, strain, management practices, and lighting, all of which can affect the growth and live performance of future placement chicks, their processing yield, and ultimately the occurrence of breast myopathies [52–56]. Environmental factors during the incubation period can also affect muscular physiology. For instance, increased temperatures during late incubation, as well as mid or late hatching, have been shown to reduce the severity of myodegeneration in broilers later in life [57]. Another study documented that WB was more prevalent in broilers hatched from eggs stored longer (8 to 14 d), compared to short storage (1 to 7 d) before incubation [45]. Detailed evaluation of how factors associated with hatchery and breeder flocks may influence development of breast myopathies is warranted in future studies.

In our study, unconditional association analysis showed that the odds of having SM was 2.25 times higher in female broilers, while the odds of having WB was 1.56 times higher in males (S2 Table). Pascual et al. [58] also reported that females had a higher prevalence of SM. During our stepwise regression analyses using the likelihood ratio test, however, the *sex* variable was removed from the final multivariable regression models of both SM and WB. This is likely due to unknown functional relationships between *sex* and other independent variables, when explaining SM and WB occurrence [59]. Aguirre et al. [31] also reported that *sex* was not a significant variable for breast myopathies, although raw frequency indicated that male broilers had more frequently WB and WS.

It has been also suggested that nutrition and genetics affect the occurrence of breast myopathies [19, 60]. In the present study, all flocks were fed with a corn-based diet and had the same genetic background (Ross 708), and therefore, these variables were not used to build the multivariable model.

#### Flock health variables

Flocks that received *coccidiosis vaccine* and/or *infectious bursal disease vaccine* had a higher risk of SM, while flocks that did not receive *coccidiosis vaccine* had a higher risk of WB. Vaccination might have affected the initial growth performance of the flocks, even though final weight at slaughter was similar between treated and untreated ones. Gautier et al. [61] reported that, up to 20 d, weight gain and feed intake were lower in broilers that received the coccidiosis vaccine compared to those that did not. Lee et al. [62] also showed that vaccination against coccidia had a negative impact on weight gain of broilers between 22 to 28 d. Therefore, it is possible that an initial decrease in the flock’s growth rate due to vaccination may influence development of myopathies. This is supported by Meloche et al. (2018), who reported that lighter broilers during the first stage of the rearing period had decreased prevalence of WB at slaughter [27]. Similarly, our logistic regression analysis showed an almost 2-fold increased odds of developing WB in non-vaccinated broilers compared to vaccinated ones. This effect, however, is likely not to be the same for all vaccines, as vaccination schedules and the response to different antigens varies [63]. For instance, administration of the *infectious tenosynovitis vaccine* was not associated with myopathy development, in our study. While growth performance could be the underlying reason for the associations between vaccination and myopathies, it remains unclear why SM and WB would present positive and negative associations, respectively, with coccidiosis vaccination.

The occurrence of WB was associated with *mortality rate during grow-out*, but the mechanism of the association is not clear. Mortality rate is affected by overall health, housing factors and management routines including flock size, stocking density, type of ventilation, drinking and heating systems [64]. The median mortality rate during grow-out reported in our study (i.e., 2.33%) is similar to what was reported by Torrey et al. (i.e., 2.52%) [65]. In this study, the authors reported that total mortality did not differ between conventional and slow-growing strains, even though conventional strains showed the greatest incidence of myopathies. Overall, other hidden factors, broadly associated with differing management practices, could affect the relationship with the occurrence of breast myopathies. Moreover, it should be considered that mortality rate during grow-out is a proxy variable for development of myopathies, since breast fillets were not likely assessed from birds with severe health issues, which would have been culled before slaughter. Further work is required to identify the association between mortality rate during grow-out and the occurrence of breast myopathies.

#### Transportation and processing plant variables

The occurrence of SM was affected by the *truck travel time* and *hold time on lairage at plant*, whereas the occurrence of WB was affected by *birds per crate*, *DOA*, *loading duration*, and *hold time on lairage at plant*. The relationship between these variables and the occurrence of SM or WB is unclear, and not mechanistically defined. In our cohort, the reported time interval from feed withdrawal to deboning was no more than 24 hrs, and this time is too short to significantly alter the tissue structure of the pectoral muscle. While scarring (fibrosis) in the skeletal muscle develops between 1 to 3 weeks after the initial injury [66, 67], it has also been suggested that increased corticosterone levels (as seen in stressful events) shortly before slaughter may affect meat quality through increased proteolysis or altered development of *rigor mortis* [68, 69]. Increased proteolytic activity is mediated mainly through matrix metalloproteinases (MMPs), which include enzymes such as collagenases and gelatinases that play a role in the degradation of structural components of the connective tissue [70]. Stress can deplete glycogen and ATP reserves in the myofibers, leading to higher final pH and softer meat [71]. It is possible that pre-slaughter stressors might have affected certain visual and tactile characteristics of the fillets, affecting our classification of myopathies. For instance, altered development of *rigor mortis* might have affected our ability to accurately classify WB, especially in the milder phenotype (WB1).

Unconditional association showed that SM was approximately 1.5 times more likely to occur in the processing plant employing the water-chill, compared to the air-chill method. Mechanical agitation of water during water-chilling using an auger increases moisture absorption [72]. It is possible, therefore, that increased water in the interstitium may promote myofiber separation, leading to increased frequency of SM. However, the *chilling method* variable was eliminated during backward elimination process using the likelihood ratio test, and was not included in the final multivariable models for SM or WB. This was likely caused by unknow functional relationship between the chilling method and other variables in explaining the frequency of SM and WB.

#### Environmental variables

A higher *temperature during grow-out* was positively correlated with the occurrence of SM, and WB had higher odds of occurring when fillets were sampled in the summer *season*. Environmental temperature can affect the physiology of the breast muscle. For instance, broilers experiencing high temperatures show different protein profiles in the breast muscle and variations in meat quality [69]. In turn, broilers with a rapid growth rate and high breast yields have reduced thermoregulatory capacity, making them vulnerable to thermal challenges [73]. Thus, it appears that higher temperature contributes to the development of SM and WB, although the mechanism of this association remains to be elucidated. The highest tier of *temperature during grow-out* category included average temperatures between 15.8°C and 22.7°C, while the overall temperature during the summer months spanned between 11.7°C (June 2019) and 22.7°C (August 2019). Considering that summer in Canada is not as hot compared to other countries with large poultry industries (such as Brazil or the southern USA), our broiler flocks were not exposed to extreme heat during the grow-out, and we did not further stratify the summer temperatures of our study.

In our model, *precipitation during grow-out* was removed during stepwise multivariable regression analysis. Relative humidity has been shown significantly to influence birds’ capacity to thermoregulate [74], and therefore the thermal-humidity index has been used to assess the impact of humidity and temperature on the growth performance of broilers [75]. It will be useful if future research will be done to investigate the association between the thermal-humidity index and the occurrence of breast myopathies.

The ICC values at the flock level of clustering were relatively small (< 1.8%), and the between-flock variance was relatively low compared to the within-flock variance. Low variability between the flocks could be explained by the uniformity of the broiler flocks included in our cohort, especially considering the similar target live weight, which is one of the most important predictor for SM and WB development [32]. The ICC values for SM were slightly higher than those of WB, indicating a greater variation in prevalence of SM compared to WB, especially at the single-bird level. This may indicate different mechanisms for development of WB and SM, even though they occur together macroscopically.

## Conclusions

This study provides a specific Canadian perspective on broiler breast myopathies. Mild WS appears to be the “new normal”, and more than one-third of breast fillets presented SM. Severe WB was observed in more than 10% of fillets, and often co-occurred with others. This high prevalence is likely to cause substantial economic losses to the Canadian poultry industry, due to decreased consumer acceptance and fillet depreciation.

In agreement with other studies, our regression analysis models showed that the development of breast myopathies is complex and multi-factorial, and it is influenced by demographics, health, husbandry, and even external environmental factors. In our study, five risk factors were shared between SM and WB; including positive association with live weight at slaughter and high temperature during grow-out. Nonetheless, three risk factors showed opposite effects on the odds of development of these myopathies, such as coccidiosis vaccination. This is surprising, considering that approximately 36% of the fillets in our cohort had both SM and WB, and suggests that WB and SM are distinct myopathies that do not necessarily share the same pathogenesis. We propose that SM could be caused by disruption of the muscle at the processing plant, where mechanical stresses act on a weakened scaffold. This is a different mechanism compared to WB, and it could explain the discrepancies in significant risk factors found in our study. Further studies are needed to determine the relevance and nature of such associations (i.e., mechanisms), so that targeted mitigating strategies can be implemented. Despite the complexity of our findings, however, it seems that the occurrence of these myopathies could be reduced by growing broilers to less than 2.46 kg, rather than encouraging excessive weight gain.

## Supporting information

S1 Table Macroscopic scoring scheme of breast myopathies. (PDF)

S2 Table Descriptive statistics and unconditional associations of variables for the occurrence of breast meat myopathies in Ontario, Canada (2019–2020) are listed for each predictor variable. (PDF)

S1 Fig Categorization of multiple time segments (durations) associated with flock transportation from the farm and the beginning of the deboning process. (PDF)

S2 Fig Flow diagram demonstrating the statistical model building process of variable selection and stepwise logistic regression analysis for spaghetti meat and woody breast. (PDF)
