# Supplementary material for: Prevalence of breast muscle myopathies (spaghetti meat, woody breast, white striping) and associated risk factors in broiler chickens from Ontario Canada
Source: PLoS One. 2022 Apr 15;17(4):e0267019. doi: 10.1371/journal.pone.0267019 (PMC9012353; doi:10.1371/journal.pone.0267019)
Supplement: S1 Table — (PDF) [file pone.0267019.s001.pdf]

S1 Table. Macroscopic scoring scheme of breast myopathies

Spaghetti meat (SM)	**SM0 (absent):** without any myofiber separation<br>**SM1 (present):** distinct myofiber separation
Woody breast (WB)	**WB0 (absent):** without abnormal firmness<br>**WB1 (moderate):** moderate increase in firmness either in the cranial or caudal aspects of the fillets, or both<br>**WB2 (severe):** distinct increase in firmness diffusely throughout the fillets
White striping (WS)	**WS0 (absent):** no distinct white lines<br>**WS1 (mild):** 1 to 40 white lines with thickness < 1 mm<br>**WS2 (moderate):** > 40 white lines, or 1 to 5 line(s) of 1.0 mm to 1.9 mm in width<br>**WS3 (severe):** > 5 lines with the thickness of 1.0 mm to 1.9 mm or ≥ 1 lines with the thickness of 2.0 mm
