# Supplementary material for: Prevalence of breast muscle myopathies (spaghetti meat, woody breast, white striping) and associated risk factors in broiler chickens from Ontario Canada
Source: PLoS One. 2022 Apr 15;17(4):e0267019. doi: 10.1371/journal.pone.0267019 (PMC9012353; doi:10.1371/journal.pone.0267019)
Supplement: S2 Table — (PDF) [file pone.0267019.s002.pdf]

S2 Table. Descriptive statistics and unconditional associations of variables for the occurrence of breast meat myopathies in Ontario, Canada (2019 – 2020) are listed for each predictor variable.

Associations of the flock and environmental variables, related to the occurrence of the breast myopathies, for each predictor assessed within univariable logistic regression model, as related to the occurrence of spaghetti meat (SM), and woody breast (WB) as the outcome of interest

Variable	Number	Type	Value	Odds ratios for SM	P value for SM	Odds ratios for WB	P value for WB
**Farm management and spatial variables**							
FSA of hatcheries	5,250	Categorical	North	1.40	<0.001	1.02	0.671
	4,000	Categorical	South	Reference		Reference	
Number of floors in a barn	6,250	Categorical	>1 floor	0.68	<0.001	1.18	0.004
	3,000	Categorical	1 floor	Reference		Reference	
**Flock demographics variables**							
Age (d)	9,250	Continuous	36 - 43	1.17	<0.001	0.95	0.001
Average live weight at slaughter (g)	3,250	Categorical	Low (1,940 – 2,290)	Reference		Reference	
	3,000	Categorical	Medium (2,300 – 2,450)	1.26	<0.001	0.89	0.072
	3,000	Categorical	High (2,460 – 2,630)	1.16	0.005	1.34	<0.001
Sex	3,750	Categorical	Female	2.25	<0.001	Reference	
	2,750	Categorical	Male	Reference		1.56	<0.001
	2,750	Categorical	Mixed	1.45	<0.001	1.04	0.499
Source of chicks	7,250	Categorical	Domestic	Reference		Reference	
	1,000	Categorical	Mixed	1.38	<0.001	0.53	<0.001
	1,000	Categorical	USA	1.49	<0.001	0.78	0.002

S2 Table continued

Variable	Number	Type	Value	Odds ratios for SM	P value for SM	Odds ratios for WB	P value for WB
**Flock health**							
Disease observed	8,000	Categorical	Disease not observed	Reference		Reference	
	1,250	Categorical	Disease observed	2.30	<0.001	0.90	0.186
Mortality rate during grow-out (%)	3,250	Categorical	Low (1.25–2.0)	Reference		Reference	
	4,250	Categorical	Medium (2.1–3.0)	1.09	0.082	0.75	<0.001
	1,750	Categorical	High (3.1–5.8)	1.16	0.014	0.67	<0.001
Raised without antibiotics (RWA)	7,250	Categorical	Non-RWA	Reference		Reference	
	2,000	Categorical	RWA	1.32	<0.001	0.82	0.003
Coccidiosis vaccine	1,500	Categorical	Administered	1.33	<0.001	0.75	<0.001
	7,750	Categorical	Not administered	Reference		Reference	
Infectious bursal disease vaccine	8,000	Categorical	Administered	1.75	<0.001	1.04	0.599
	1,250	Categorical	Not administered	Reference		Reference	
Infectious tenosynovitis vaccine	1,750	Categorical	Administered	1.13	0.021	1.00	0.986
	7,500	Categorical	Not administered	Reference		Reference	
Marek's disease vaccine	8,500	Categorical	Administered	0.97	0.585	0.94	0.420
	750	Categorical	Not administered	Reference		Reference	
Category III antimicrobials	6,250	Categorical	Administered	0.96	0.326	1.09	0.145
	3,000	Categorical	Not administered	Reference		Reference	
Coccidiostats and/or ionophores	6,750	Categorical	Administered	0.71	<0.001	1.19	0.002
	2,500	Categorical	Not administered	Reference		Reference	

S2 Table continued

Variable	Number	Type	Value	Odds ratios for SM	P value for SM	Odds ratios for WB	P value for WB
**Transportation variables**							
Birds per crate	5,750	Categorical	Low (8–9)	Reference		Reference	
	500	Categorical	Medium (9.5–10)	1.35	0.001	1.02	0.854
	3,000	Categorical	High (10.5–12)	0.63	<0.001	1.46	<0.001
Dead on arrival (%)	9,250	Continuous	0–0.16	0.96	<0.001	1.03	<0.001
Duration without feed (min)	3,250	Categorical	Low (300–685)	Reference		Reference	
	3,000	Categorical	Medium (686–762)	0.87	0.008	1.03	0.646
	3,000	Categorical	High (763–1005)	0.83	0.001	0.88	0.056
Duration without water (min)	9,250	Categorical	Low (113–271)	Reference		Reference	
	9,250	Categorical	Medium (272–363)	0.97	0.572	1.14	0.053
	9,250	Categorical	High (364–470)	0.99	0.921	0.98	0.801
Loading duration (min)	9,250	Categorical	Low (40–75)	Reference		Reference	
	9,250	Categorical	Medium (76–100)	0.99	0.789	0.95	0.385
	9,250	Categorical	High (101–150)	1.05	0.317	1.29	<0.001
Truck travel time (min)	3,250	Categorical	Low (20–68)	Reference		Reference	
	3,000	Categorical	Medium (69–163)	1.28	<0.001	0.83	0.006
	3,000	Categorical	High (164–798)	0.79	<0.001	0.97	0.683
**Processing plant variables**							
Chilling method	4,750	Categorical	Air-chill	Reference		Reference	
	4,500	Categorical	Water-chill	1.45	<0.001	0.91	0.096
Condemnation (%)	3,250	Categorical	Low (0.01–0.45)	Reference		Reference	
	3,000	Categorical	Medium (0.46–1.14)	0.94	0.211	1.04	0.590
	3,000	Categorical	High (1.15–2.81)	0.81	<0.001	1.25	0.001
Hold time on lairage at plant (min)	3,250	Categorical	Low (10–110)	Reference		Reference	
	3,000	Categorical	Medium (111–160)	1.17	0.002	0.79	0.001
	3,000	Categorical	High (161–269)	0.77	<0.001	0.78	<0.001

S2 Table continued

Variable	Number	Type	Value	Odds ratios for SM	P value for SM	Odds ratios for WB	P value for WB
**Environmental and temporal variables**							
Temperature during grow-out (ºC)	3,250	Categorical	Low (-9.8–0)	Reference		Reference	
	3,250	Categorical	Medium (0.1–15.7)	2.00	<0.001	0.61	<0.001
	2,750	Categorical	High (15.8–22.7)	2.33	<0.001	0.58	<0.001
Temperature during transport (ºC)	3,250	Categorical	Low (-9.8–2.9)	Reference		Reference	
	3,000	Categorical	Medium (3.0–12.9)	1.65	<0.001	0.60	<0.001
	3,000	Categorical	High (13.0–23.7)	2.09	<0.001	0.70	<0.001
Season	1,250	Categorical	Spring	1.56	<0.001	3.85	<0.001
	2,500	Categorical	Summer	2.44	<0.001	1.52	<0.001
	2,750	Categorical	Fall	1.99	<0.001	0.89	<0.001
	2,750	Categorical	Winter	Reference		Reference	
Precipitation during grow-out (mm)	3,250	Categorical	Low (0–66)	Reference		Reference	
	3,000	Categorical	Medium (67–100)	1.60	<0.001	0.89	0.074
	3,000	Categorical	High (101–165)	1.35	<0.001	1.05	0.466
Precipitation during transport (mm)	3,250	Categorical	Low (0–0.1)	Reference		Reference	
	3,000	Categorical	Medium (0.2–1.7)	0.80	<0.001	1.06	0.363
	3,000	Categorical	High (1.8–20.2)	0.67	<0.001	0.99	0.979
