# Supplementary material for: Prevalence of breast muscle myopathies (spaghetti meat, woody breast, white striping) and associated risk factors in broiler chickens from Ontario Canada
Source: PLoS One. 2022 Apr 15;17(4):e0267019. doi: 10.1371/journal.pone.0267019 (PMC9012353; doi:10.1371/journal.pone.0267019)
Supplement: S1 Fig — (PDF) [file pone.0267019.s003.pdf]

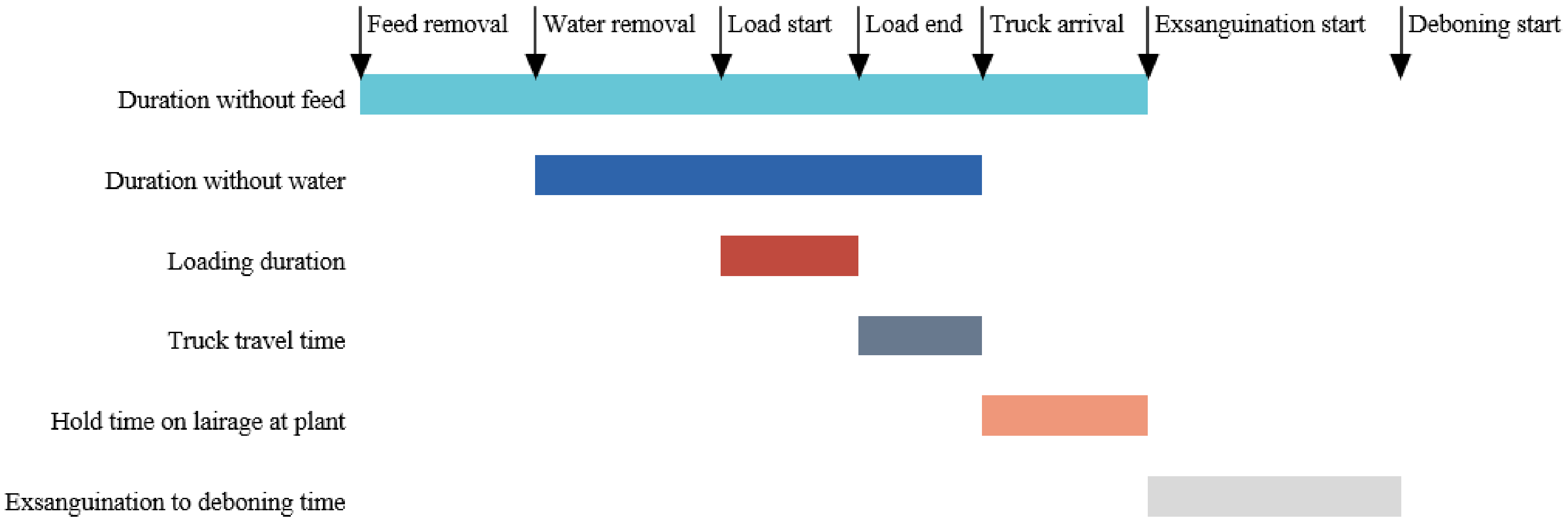

S1 Figure Categorization of multiple time segments (durations) associated with flock transportation from the farm and the beginning of the deboning process.
