# Supplementary material for: Prevalence of breast muscle myopathies (spaghetti meat, woody breast, white striping) and associated risk factors in broiler chickens from Ontario Canada
Source: PLoS One. 2022 Apr 15;17(4):e0267019. doi: 10.1371/journal.pone.0267019 (PMC9012353; doi:10.1371/journal.pone.0267019)
Supplement: S2 Fig — (PDF) [file pone.0267019.s004.pdf]

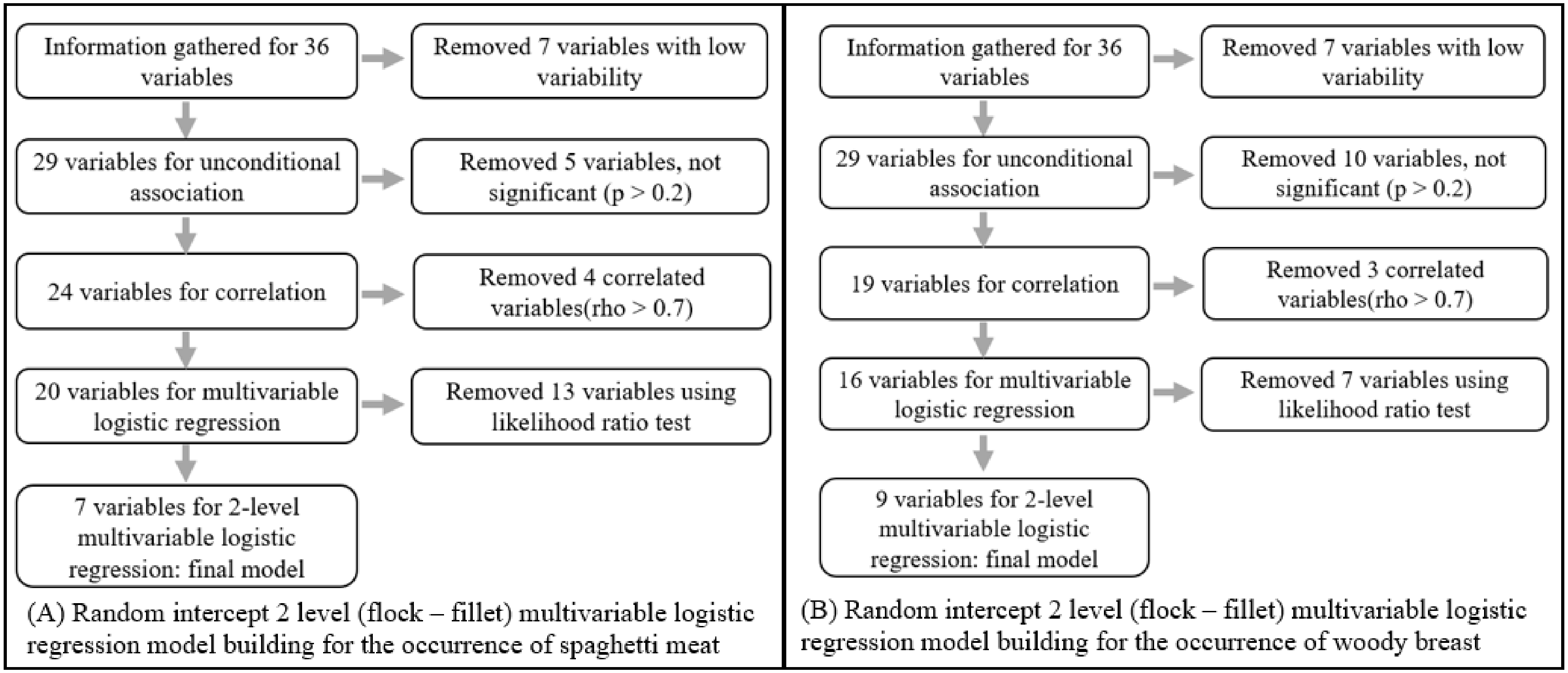

S2 Figure. Flow diagram demonstrating the statistical model building process of variable selection and stepwise logistic regression analysis for spaghetti meat and woody breast.

(A) Random intercept 2-level (flock-fillet) multivariable logistic regression model building for the occurrence of spaghetti meat: information on 36 variables was collected from processing plants and Environment Canada weather stations. Seven variables with low variability (< 5.5%) were removed, 29 variables were used for unconditional associations, and 5 variables were removed using a relaxed p-value ($p < 0.2$). Twenty-four variables were used for the correlation analysis, and 4 variables were removed because of strong collinearity (rho > 0.7). Multivariable logistic regression models were built for 20 variables. Thirteen variables were removed using the likelihood ratio (LR) test during a manual backward elimination process. Random intercept 2-level (flock-fillet) model was built, an intra-class correlation coefficient (ICC) was calculated, and the final model included 7 variables.

(B) Multivariable logistic regression model building for the occurrence of the woody breast: information on 36 variables was collected from processing plants and Environment Canada weather stations. Seven variables with low variability (< 5.5%) were removed, 29

variables were used for unconditional associations, and 10 variables were removed using a relaxed p-value ($p < 0.2$). Nineteen variables were used for the correlation analysis, and 3 variables were removed because of strong collinearity (rho > 0.7). Multivariable logistic regression models were built for 16 variables. Seven variables were removed using the LR test during a manual backward elimination process. Random intercept 2-level model was built, an ICC was calculated, and the final model included 9 variables.
